# Primary Adult Renal Ewing’s Sarcoma: A Rare Entity

**DOI:** 10.7759/cureus.22302

**Published:** 2022-02-16

**Authors:** Darilin M Shangpliang, Gordon Rangad, Jayanta Kumar Das, Kirtijit Chakma

**Affiliations:** 1 Department of Pathology, Nazareth Hospital, Shillong, IND; 2 Department of General and Minimally Invasive Surgery, Nazareth Hospital, Shillong, IND

**Keywords:** primitive neuroectodermal tumors, ewing sarcoma (es), immunohistochemistry, adult, renal

## Abstract

Ewing’s sarcoma/primitive neuroectodermal tumors are high-grade small round blue cell tumors traditionally found in children and adolescents.These tumors primarily affect the bone and soft tissue, with extraskeletal sites rarely being affected. The clinical presentation and imaging findings are non-specific and are not characteristic. The diagnosis is essentially based on the histopathologic findings assisted by immunohistochemistry and/or cytogenetic molecular studies. Proper diagnoses and timely management of this tumor are essential owing to the aggressive nature and poor prognosis of the disease.

## Introduction

Ewing’s sarcoma (ES)/primitive neuroectodermal tumor (PNETs) are high-grade small round blue cell tumors typically found in children and adolescents [1]. The most common site of primary disease is bone, though extraskeletal primary sites are known to occur and can affect the skin, soft tissue, or viscera. Primary renal presentation is outstandingly rare, and confusion with other common primary small round cell tumors of childhood such as Wilm’s tumor and neuroblastoma is not uncommon. One must also rule out other small round cell tumors such as lymphoma, monophasic synovial sarcoma, and desmoplastic small round cell tumor presenting in the adult population, which often leads to delay in diagnosis and misdiagnosis [2]. ES of primary renal origin is exceedingly rare and is associated with a poor prognosis and a high propensity for metastasis [3].

## Case presentation

**Figure 1:**
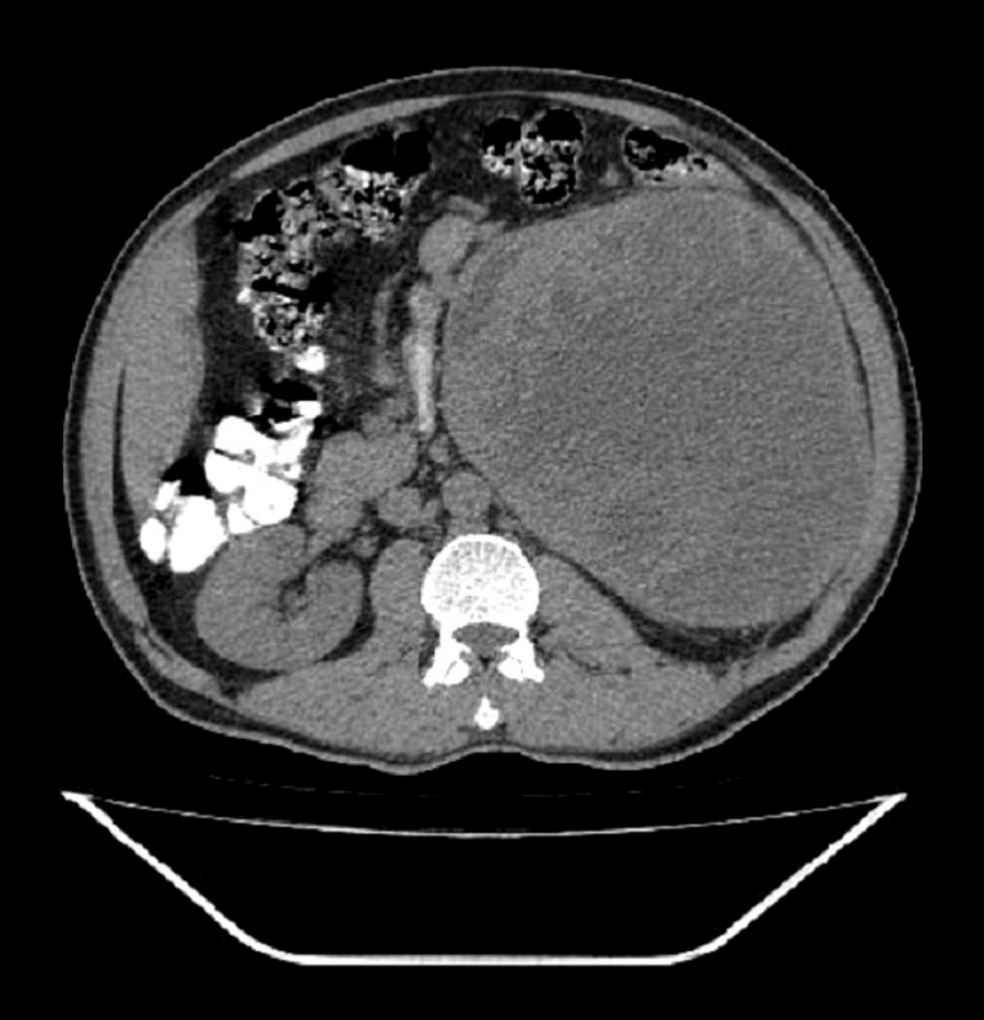
CECT of the abdomen showing a large solid cystic lobulated mass of the left kidney measuring 26 x 16cm in size with perilesional streakiness. CECT, contrast-enhanced computed tomography

A 45-year-old man presented with left upper abdominal swelling since one year, and he subsequently noticed swelling of his left lower limb on standing. There was no other significant medical history. On physical examination, a large and hard lump was felt on the left hypochondrium and left lumbar region. The lump was bimanually palpable with no extension across the midline. All the routine blood investigations including serum creatinine were normal. Urine analysis was normal. A contrast-enhanced computed tomography (CECT) scan showed a large, solid cystic, lobulated mass (26.0 x 16cm) with perilesional streakiness arising from the left kidney with compression of surrounding structures, suggestive of malignancy (Figure 1). The patient underwent radical left nephrectomy.

**Figure 2:**
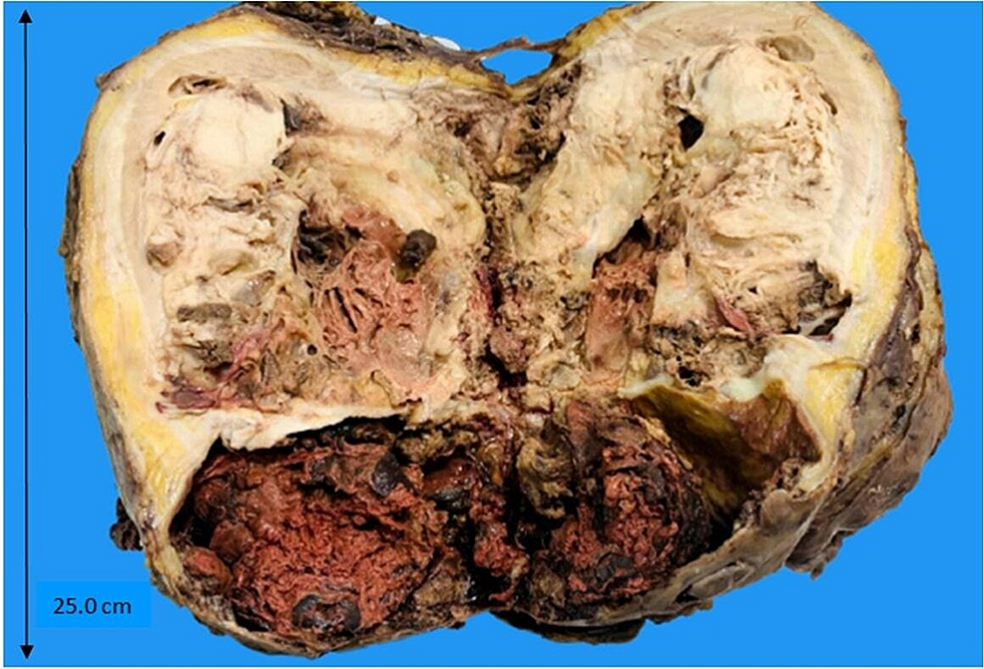
Gross image showing cut surface of nephrectomy specimen showing variegated growth with large areas of hemorrhage and necrosis.

Grossly, the kidney weighed 2.5kg and measured 25.0 x 18.0 x 10.5cm. External surface shows attached perinephric fat. The cut surface shows a variegated growth involving the entire kidney. The growth was friable with areas of hemorrhage and necrosis involving both the cortex and medulla. The corticomedullary junction, pelvicalyceal system, and renal parenchyma were effaced, with invasion of the renal sinus (Figure 2).

**Figure 3:**
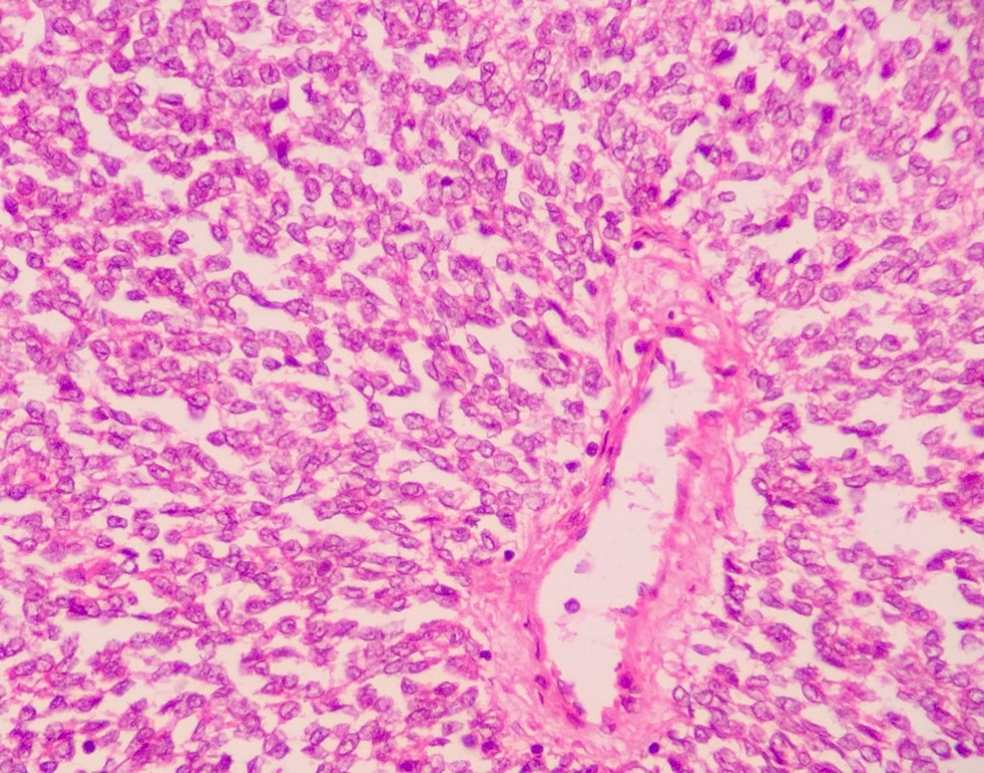
Hematoxylin and eosin stain at 400x magnification showing small round nuclei with finely dispersed chromatin and scant ill-defined cytoplasm.

**Figure 4:**
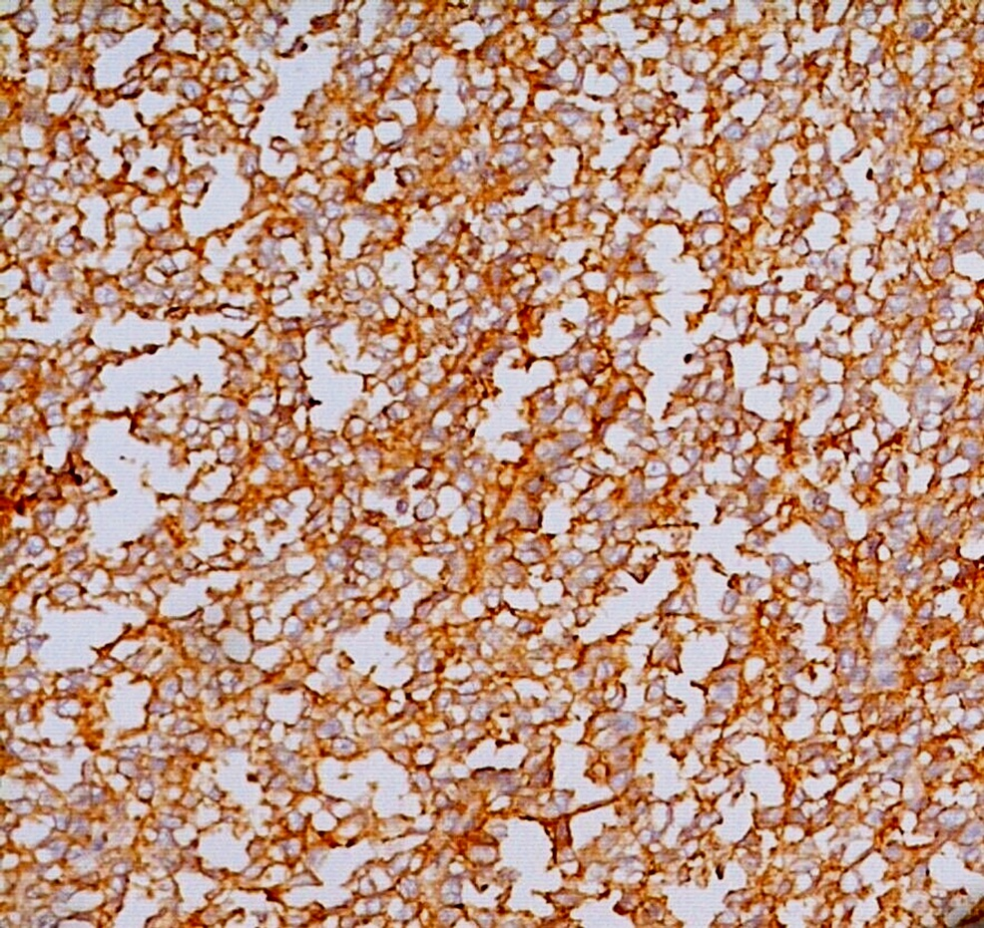
IHC showing diffuse membranous positivity of tumor cells for CD99 (400x). IHC, immunohistochemistry

Microscopically, the tumor revealed a well-circumscribed lesion surrounded by a pseudocapsule. The tumor was markedly cellular and composed of sheets of small round cells with indistinct cell borders and uniform round nuclei, showing dispersed chromatin and chromatin clearing. Nucleoli were absent to inconspicuous at 400x magnification. Fine fibrovascular cores, vague papillary pattern, and a few scattered pseudorosettes were noted (Figure 3). Several mitotic figures with large areas of hemorrhage and necrosis were noted. Glycogen was highlighted in tumor cells on periodic acid-Schiff staining. On immunohistochemistry (IHC), the tumor cells were strongly positive for CD99, with patchy positivity for NKX2.2 and synaptophysin and negative for cytokeratin (CK), epithelial membrane antigen (EMA), leukocyte common antigen (LCA), and Bcl2 antibodies, thus confirming a diagnosis of ES/PNET (Figure 4). The patient was then initiated on treatment with multi-agent chemotherapy. He is presently doing well and has put on weight after seven months of follow-up.

## Discussion

ES and PNETs were formerly classified as two distinct pathologic entities, albeit they both share common stem cell precursor and distinct chromosomal abnormality. Owing to their similar histologic and cytogenic characteristics, these tumors are now categorized under a spectrum of neoplastic diseases now known as Ewing’s sarcoma family tumors [2].

ES is an aggressive osseous and soft tissue tumor in children and young adults. Even though primary ES of the retroperitoneum, pelvis, and visceral organs is rare, they have been described in the literature [4]. The first case of primary ES of the kidney was reported by Seemayer et al. in 1975. Sources of renal ES include neural cells that invaginate into the kidney during development. However, other authors theorize on the fact that embryonic neural crest cells migrate into the kidney and undergo tumorigenesis [5].

Primary ES of the kidney typically manifests in children and adolescents with a strong male predilection [4]. The clinical presentations of these patients include flank/abdominal pain, palpable mass, and hematuria [6]. Usually, they remain asymptomatic for a long time, until the mass reaches a palpable size, as seen in our case. These tumors usually arise from the medullary or pelvic region and can involve the entire kidney with invasion into the renal vein and inferior vena cava [4]. In our case, the tumor extensively involved the entire left kidney with compression of the common iliac vein, accounting for the swelling of the left lower limb.

No specific signs of ES/PNETs have been described radiologically. The imaging characteristics of renal sarcoma are indistinguishable from other renal tumors [2]. The major challenge in the diagnosis of this entity is its morphologic similarity with other small round blue cell tumors. In children, one must rule out Wilm’s tumor, neuroblastoma, and primary renal rhabdomyosarcoma. In the adult age group, the main differential diagnoses are non-Hodgkin’s lymphoma, monophasic synovial sarcoma, and desmoplastic small round cell tumor [7].

The diagnosis of ES/PNET is strongly based on the pathologic findings assisted by IHC and/or molecular analysis. A combination of IHC markers such as CD99, FLI-1, and NKX2.2 can help in the diagnosis [3]. In the present case, the possibility of lymphoma was excluded on the basis of extensive presence of pseudorossettes coupled with the negative staining for LCA. The absence of immunostaining for bcl-2 has helped in ruling out the diagnosis of monophasic synovial sarcoma. Desmoplastic small round cell tumor was ruled out on the basis of the negative reaction for CK and EMA. Molecular studies play an important role in confirming the diagnosis by the demonstration of the translocation t(11: 22) (q24; q12) resulting in the production of EWS/FLI-1 fusion gene by fluorescence in-situ hybridization (FISH) technique or reverse transcriptase polymerase chain reaction (RT-PCR) [8].

There is no clear consensus for the treatment of renal ES/PNETs. However, the currently employed treatment protocol involves a combination of surgical resection, adjuvant chemotherapy, and/or radiotherapy. The standard chemotherapy regimen includes a three-drug combination of vincristine, doxorubicin, and d-actinomycin, along with the addition of alternating cycles of etoposide and ifosfamide. Radiotherapy has been considered for treating local recurrences or residual tumor. However, this treatment protocol appears to be of lesser benefit in these patients owing to advanced presentation of the disease. ES/ PNETs exhibit an aggressive course characterized by early metastatic disease [9,10]. Extraosseous ES has a poorer prognosis as compared to its osseous counterpart with a high incidence of local recurrence and distant metastasis [11]. Despite aggressive treatment, the prognosis of renal ES is poor, with an overall cure rate of 20% [2,8].

## Conclusions

Renal ES/PNETs are rare fatal tumors presenting in children and adolescents; however, one must include them in the differential diagnosis in the adult population presenting with a renal mass. The aggressive nature of this entity warrants exclusion of other malignant small round cell tumors by histomorphology, IHC, and cytogenetic studies at the earliest. Owing to its scarcity, no standard treatment protocols have been established. However, a multimodality treatment approach is implemented in view of the poor prognosis of the disease.
